# Research progress on the pharmacological activity, biosynthetic pathways, and biosynthesis of crocins

**DOI:** 10.3762/bjoc.20.68

**Published:** 2024-04-09

**Authors:** Zhongwei Hua, Nan Liu, Xiaohui Yan

**Affiliations:** 1 State Key Laboratory of Component-based Chinese Medicine, Tianjin University of Traditional Chinese Medicine, No. 10 Poyang Lake Road, Tuanbo New Town, Jinghai District, Tianjin 301617, China; 2 School of Chinese Materia Medica, Tianjin University of Traditional Chinese Medicine, No. 10 Poyang Lake Road, Tuanbo New Town, Jinghai District, Tianjin 301617, China

**Keywords:** biosynthetic pathway, crocetin, crocins, metabolic engineering, pharmacological activity

## Abstract

Crocins are water-soluble apocarotenoids isolated from the flowers of crocus and gardenia. They exhibit various pharmacological effects, including neuroprotection, anti-inflammatory properties, hepatorenal protection, and anticancer activity. They are often used as coloring and seasoning agents. Due to the limited content of crocins in plants and the high cost of chemical synthesis, the supply of crocins is insufficient to meet current demand. The biosynthetic pathways for crocins have been elucidated to date, which allows the heterologous production of these valuable compounds in microorganisms by fermentation. This review article provides a comprehensive overview of the chemistry, pharmacological activity, biosynthetic pathways, and heterologous production of crocins, aiming to lay the foundation for the large-scale production of these valuable natural products by using engineered microbial cell factories.

## Introduction

Crocins are hydrophilic apocarotenoids mainly isolated from the fruit of *Gardenia jasminoides* Ellis and the stigma tissue of *Crocus sativus* L. Crocins are the main active ingredients of *C. sativus*, a precious medicinal plant known as the "gold of spices". They are also responsible for the characteristic red color of saffron. Compounds of the crocin family are the monoglycosyl, diglycosyl, or triglycosyl products of 8,8′-diapocarotene-8,8′-dioic acid (crocetin, **1**) or derivatives thereof. In addition to *C. sativus* and *G. jasminoides*, *Buddleja officinalis* and *Buddleja davidii* from the Loganiaceae family [1–2], *Nyctanthes arbor-tristis* from the Oleaceae family [3], and *Arctium lappa* from the Compositae family also contain crocins [4]. The content of crocins varies significantly among different plant species and different organs of the same plant.

Crocins are utilized extensively as colorants and flavoring agents in the food, cosmetics, and pharmaceutical industries [5]. They exhibit a broad spectrum of pharmacological activity, including antitumor [6], anti-inflammatory [7], hepatoprotective [8], nephroprotective [9], antidepressant [10], antioxidant [11], and antidiabetic [12] properties. The Saffron Multi-glycoside Tablet (Xihonghua Zonggan Pian) ameliorates myocardial ischemia or myocardial infarction induced by coronary ligation. Although crocin and crocetin derivatives exhibit a diverse scope of biological activity, they are characterized by poor stability and low bioavailability, which has significantly impeded clinical developments. Recently, novel pharmaceutical systems, such as liposomes, microcapsules, and nanoparticles, were adopted to enhance the stability and bioavailability of these compounds [13–15].

To find a sustainable way of supplying crocins, researchers have developed various approaches to produce them, including plant cell suspension culture, heterologous biosynthesis, and total synthesis [16–17]. Crocins can be obtained from plant cell culture, but the production is prone to epigenetic silencing and toxic intermediates. The chemical synthesis of crocins is challenging due to the presence of numerous chiral centers, which complicates matching supply to demand. Engineering microbial strains for the heterologous production of rare natural products has emerged as a promising approach [18–19]. With the elucidation of the biosynthetic pathways for crocins in plants, the heterologous production of crocin and crocetin derivatives in microorganisms has been achieved by various teams.

This article comprehensively reviews the research progress on the extraction, separation, pharmacological activity, biosynthesis, and synthetic biology of crocins. The biosynthesis of crocins is depicted in detail to shed light on the efficient heterologous production of crocins in microorganisms to break the current bottleneck of a sustainable supply. It provides the basis for the further development of crocins in the food, cosmetic, and pharmaceutical industries.

## Review

### Chemical properties and distribution of crocins

**Figure 1:**
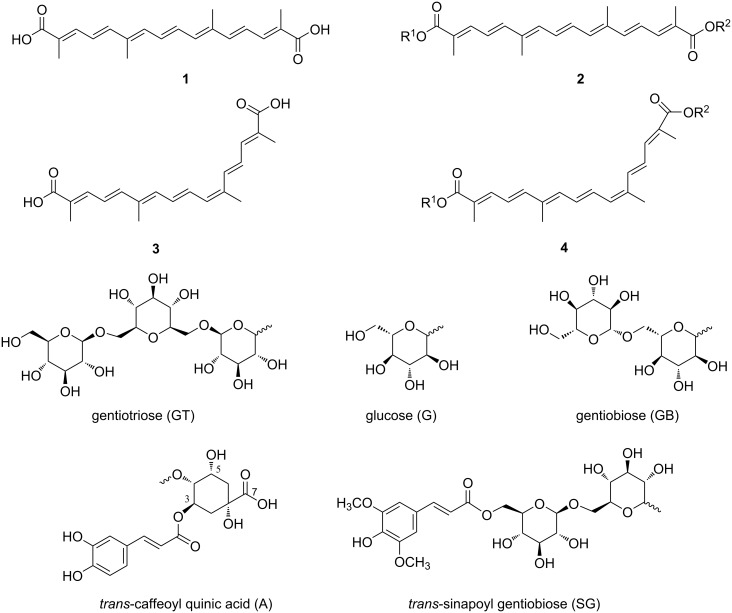
Principal structure of crocin and crocetin derivatives, including common substituents of the crocetin skeleton.

More than ten crocins have been identified to date (Table 1). Crocin-I (**2a**), crocin-II (**2b**), crocin-III (**2c**), crocin-IV (**2d**), and crocin-V (**2e**) are the most common crocins, among which **2a** is the most abundant in nature. Compound **2a** is a diester derivative of crocetin (**1**), with a gentiobiose unit attached to each carboxyl group. Crocin-II (**2b**) consists of one glucose and one gentiobiose unit, crocin-III (**2c**) comprises two glucose molecules, crocin-IV (**2d**) contains only one gentiobiose moiety, and crocin-V (**2e**) has only one glucose moiety (Figure 1 and Table 1). Pure crocins form reddish-brown acicular crystals with a slight odor. They are highly soluble in hot water but slightly soluble in anhydrous ethanol and ether. The presence of multiple conjugated double bonds in crocins makes them susceptible to degradation when exposed to certain conditions, such as high temperature, the presence of metal ions or light, certain pH values, etc. For example, during the harvest of *C. sativus*, a high drying temperature leads to the cleavage of the glycosidic bonds [20–22]. Crocins are stable in alkaline and neutral solutions but are labile under acidic solutions.

**Table 1:** Structure of crocin and crocetin derivatives. A, SG, G, GB, and GT represent the common substituents of the crocin skeleton shown in Figure 1.

compound	R^1^	R^2^	molecular formula

crocetin (**1**)	H	H	C_20_H_24_O_4_
crocin-I (**2a**)	GB	GB	C_44_H_64_O_24_
crocin-II (**2b**)	G	GB	C_38_H_54_O_19_
crocin-III (**2c**)	G	G	C_32_H_44_O_14_
crocin-IV (**2d**)	H	GB	C_32_H_44_O_14_
crocin-V(**2e**)	G	H	C_26_H_34_O_9_
crocin-6 (**2f**)	GB	GT	C_50_H_74_O_29_
crocin-7 (**2g**)	GT	GT	C_56_H_84_O_34_
*trans*-crocin-4' (**2h**)	G	GT	C_44_H_64_O_24_
neocrocin C (**2i**)	GB	A at 4′′	C_48_H_60_O_22_
neocrocin F (**2j**)	SG	H	C_43_H_54_O_18_
neocrocin G (**2k**)	SG	GB	C_55_H_74_O_28_
neocrocin H (**2l**)	GB	CH_2_CH_3_	C_34_H_48_O_14_
neocrocin J (**2m**)	β-ᴅ-xylosyl-(1→6)-β-ᴅ-glucosyl	H	C_31_H_42_O_13_
13-*cis*-crocetin (**3**)	H	H	C_20_H_24_O_4_
*cis*-crocin-2 (**4a**)	GB	H	C_32_H_44_O_14_
*cis*-crocin-3 (**4b**)	GB	G	C_38_H_54_O_19_
13-*cis*-crocin (**4c**)	GB	GB	C_44_H_64_O_24_
neocrocin D (**4d**)	GB	A at 4′′	C_48_H_60_O_22_
neocrocin E (**4e**)	A at 4′′	GB	C_48_H_60_O_22_
13-*cis*-crocetin-8-*O*-β-ᴅ-glucopyranosyl- 8′-*O*-β-ᴅ-gentiobioside (**4f**)	H	GB	C_32_H_44_O_14_
13-*cis*-crocetin-8-*O*-β-ᴅ-glucopyranosyl- 8′-*O*-β-ᴅ-gentiobioside (**4g**)	G	GB	C_38_H_54_O_19_

Crocins can be detected in the flowers, fruits, stigmas, leaves, and roots of plants. The content varies significantly among different plant species and different parts within the same plant. For instance, in *C. sativus*, crocins are predominantly accumulated in the stigma, but in *G. jasminoides,* they are primarily stored in the pulp. The traditional methods for extracting crocins include ultrasound-assisted extraction (UAE), supercritical fluid extraction, enzyme-linked extraction, and microwave-assisted extraction. Among these methods, the UAE exhibits a higher extraction yield [23–24]. Recently, Fiorito et al. developed a technique that utilizes cost-effective and environmentally friendly adsorbents for the selective adsorption of crocins from an aqueous solution of saffron pollen [25].

### Pharmacological activity of crocins

**Figure 2:**
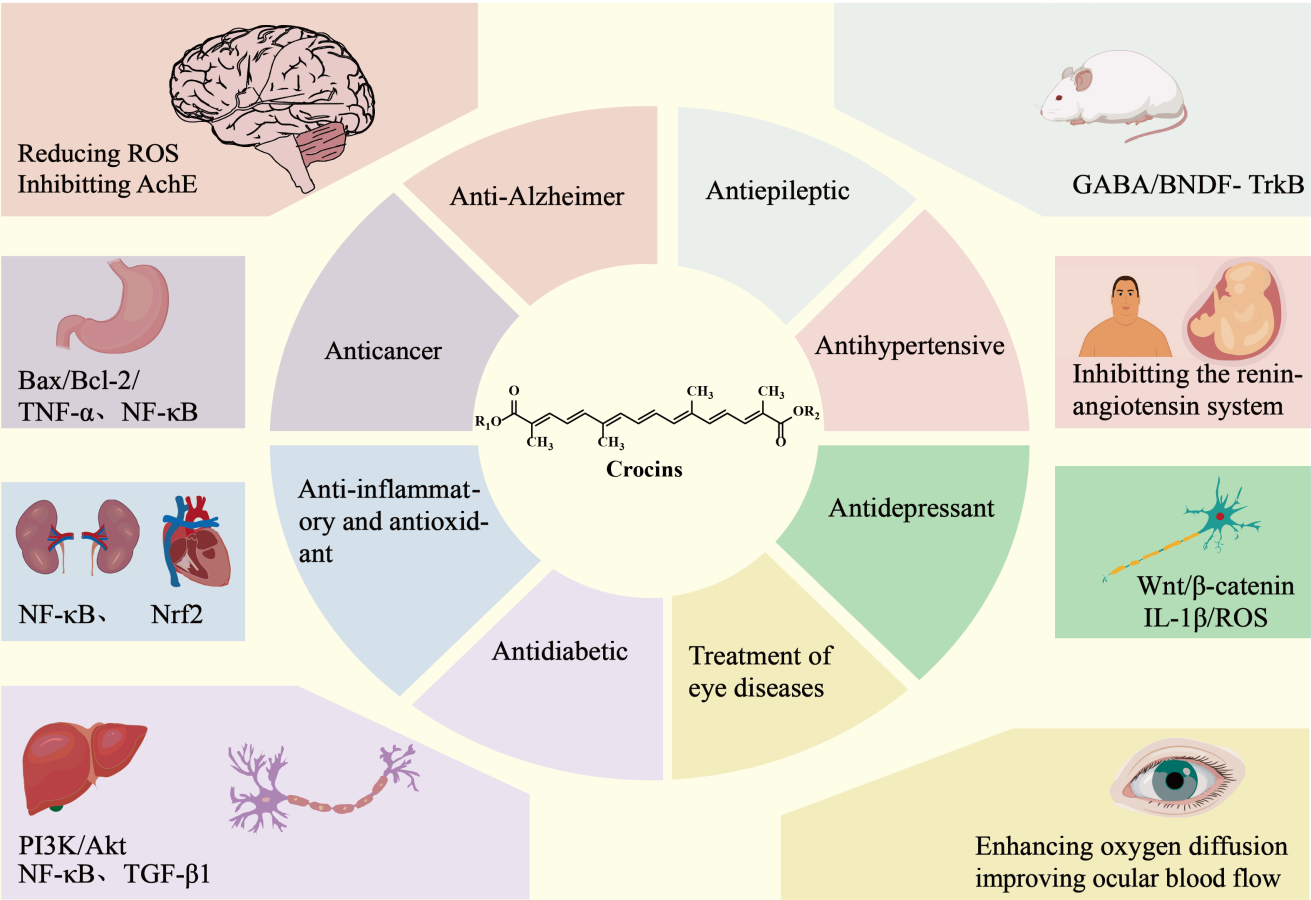
The pharmacological activity and mechanisms of action of crocins.

Various pharmacological studies have shown that crocins are effective against diseases of the central nervous system (neurodegenerative diseases, epilepsy, convulsion, and insomnia) and cardiovascular diseases (hypertension, hyperlipidemia, and atherosclerosis). In addition, they also have anticancer, anti-inflammatory, antioxidative, liver- and kidney-protective, antidepressant, and antidiabetic properties (Figure 2).

#### Neuroprotection

Pharmacokinetic investigations have shown that crocins can be hydrolyzed to crocetin (**1**) in the gastrointestinal tract of mice. Crocetin derivatives can penetrate the blood–brain barrier to exert therapeutic effects on neurodegenerative diseases, such as Alzheimer's disease, Parkinson's disease, retinal diseases, and epileptic disorders [26–27].

Four mechanisms have been reported for the treatment of Alzheimer's disease with crocins. Crocins function as antioxidants that slow down the progression of the disease by increasing the ʟ-glutathione (GSH) level and reducing the presence of reactive oxygen species (ROSs) [28]. Crocins can inhibit the activity of acetylcholinesterase and increase the acetylcholine concentration, thus improving the learning and memory ability of the brain [29]. Moreover, crocins prevent the abnormal aggregation of amyloid β-protein (Aβ), microtubule-associated protein tau, and α-synuclein (αS), thereby alleviating the apoptosis of neuronal cells and cognitive impairment [30–33]. Lastly, the extract of *C. sativus* can also improve the chronic stress-induced loss of learning and memory by reducing the ROS level or regulating the synthesis of superoxide dismutase (SOD) and glutathione peroxidase (GPx) [34].

The current clinical treatment of epilepsy relies on the use of antiepileptic drugs. However, the occurrence of drug-resistant epilepsy is a top-priority problem [35–37]. It was found that intravenous injection of 50 μg crocin could delay epileptiform activity in mice and enhance the antiepileptic effect of diazepam. The mechanism of action involved γ-aminobutyric acid (GABA) [38–40]. The brain-derived neurotrophic factor (BDNF)–tropomyosin receptor kinase B (TrkB) pathway is closely associated with epilepsy. Crocin can increase the BDNF level in the cortex of temporal lobe epilepsy and reduce the content of tumor necrosis factor α (TNF-α) in the hippocampus, thereby playing an antiepileptic role [41–42].

#### Antihypertension

Imenshahidi et al. demonstrated that crocins, safranal (**12**), and the extract of *C. sativus* can reduce the mean arterial pressure in both healthy and hypertensive rats depending on the dose [43]. In an angiotensin II-induced acute hypertension model, crocins could probably improve cardiovascular function by reducing the ROS level [44]. Chen et al. found that crocins could reduce inflammation and oxidative stress in the rat placenta and alleviate gestational hypertension. Therefore, crocins have the potential to prevent pregnancy-induced hypertension [45].

#### Anticancer

Crocins exhibit potent anticancer activity against various cancer cell lines. Jiang et al. found that crocins reduce the survival and activity of cervical cancer cells [46]. Mollaei et al. observed an increased Bax/Bcl-2 ratio in crocin-treated cancer cells. Therefore, crocins are proposed to exert anticancer activity by promoting apoptosis of cancer cells [47]. This mechanism is consistent with the results of Hoshyar et al., who observed the apoptosis-promoting activity of crocins in gastric adenocarcinoma cells [48]. In addition to the cervical cancer cell lines and gastric cancer cell lines, crocins also exhibit anticancer effects on breast, prostate, liver, colon, and leukemia cancer cell lines [49–53]. Besides regulating the Bax/Bcl-2 ratio, crocins were also reported to inhibit cell invasion and metastasis [54–55].

#### Anti-inflammation and antioxidation

Crocins exhibit anti-inflammation properties by scavenging free radicals and regulating the expression of antioxidant enzymes. Crocins can inhibit the NF-κB signaling pathway, thus downregulating the expression of inducible nitric oxide synthase (iNOS) and cyclooxygenase 2 (COX2) [11]. Crocins can also regulate the Nrf2 signaling pathway to protect the brain, liver, and other organs and decrease the expression of pro-inflammatory factors, such as TNF-α, prostaglandin E2 (PGE2), interleukin-1β (IL-1β), and interleukin-6 (IL-6) [56–58]. The combination of crocins with doxorubicin and curcumin also showed a good anti-inflammation effect [56,59].

#### Antidepression

Alsanie et al. found that the therapeutic effect of *C. sativus* extract on mild and moderate depressive disorder is comparable to antidepressants, such as imipramine and fluoxetine [10,60–61]. The antidepressant activity of *C. sativus* extract was exerted by regulating the serotonin, norepinephrine, and dopamine levels in the brain [62–64]. Further studies confirmed that crocins are the key antidepressant agents in *C. sativus* extract [65–67]. Crocins ameliorate depression-like behavior in rats by mitigating neuroinflammation and reducing IL-1β, ROS, and malondialdehyde [10,68]. Tao et al. found that crocins exert an antidepressant effect by enhancing adult hippocampal neurogenesis (AHN) levels and activating the Wnt/β-catenin pathway [69].

#### Other functions

In addition to the effects mentioned above, crocins also possess other pharmacological properties, such as antidiabetic, antiatherosclerotic, and organ-protective effects. They also have a role in immune modulation and the treatment of glaucoma and diabetic retinopathy [70–80].

#### Biosynthesis

**Figure 3:**
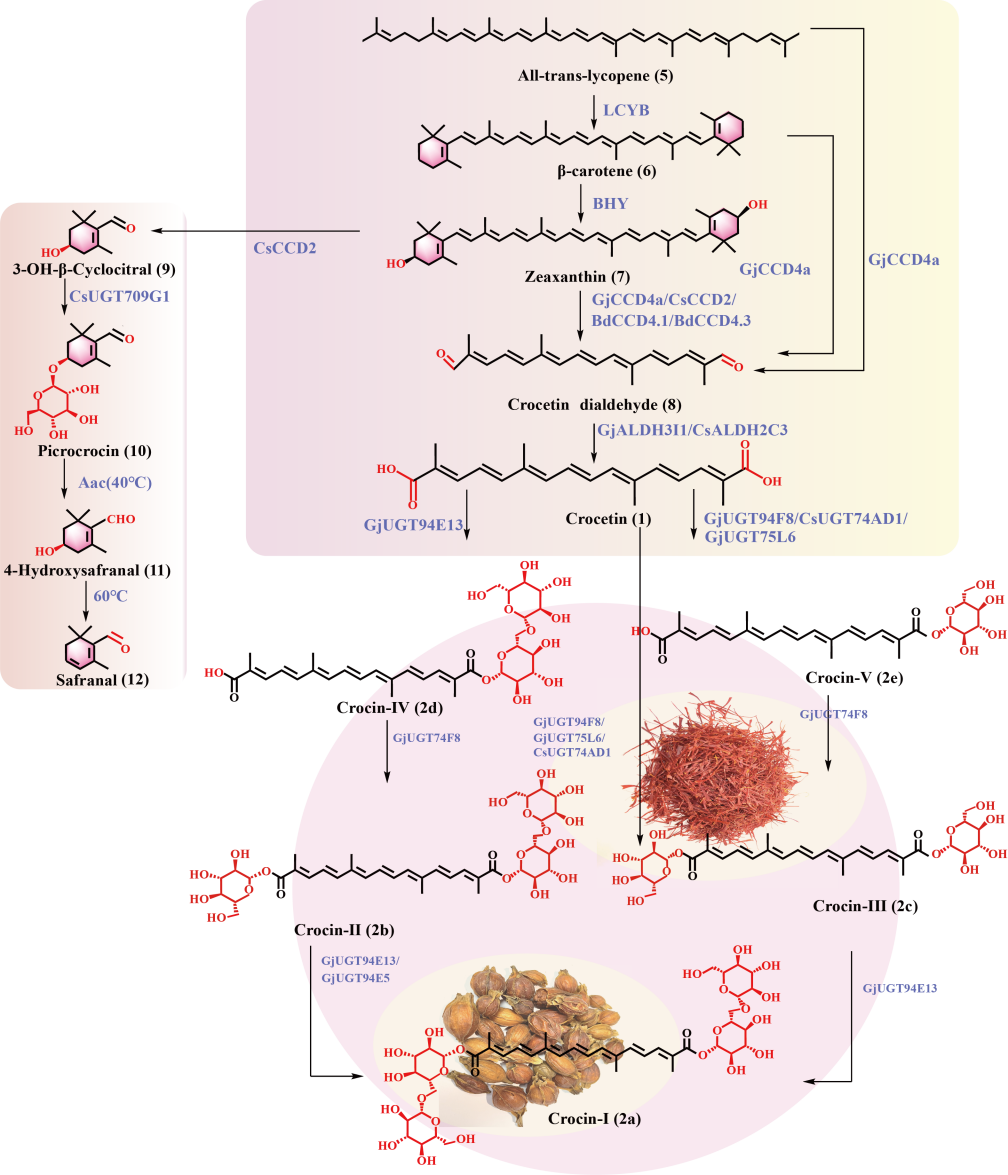
Crocin biosynthetic pathways in *C. sativus* and *G. jasminoides.* Enzyme abbreviations are as follows: lycopene β-cyclase (LCYB), β-carotene hydrolase (BHY), carotenoid cleavage dioxygenase (CCD), aldehyde dehydrogenase (ALDH), uridine diphosphate glucosyltransferase (UGT).

The biosynthetic pathways of crocins have recently been studied extensively. Crocin biosynthesis can be divided into three stages: 1) biosynthesis of lycopene (**5**) from simple carbon resources, 2) cleavage of lycopene (**5**), β-carotene (**6**), or zeaxanthin (**7**) by a carotenoid cleavage dioxygenase (CCD) to form crocetin aldehyde (**8**) and, after oxidation, **1**, and 3) glycosylation of **1** to generate crocins (Figure 3). Since the biosynthetic pathways of **5** in plants and microorganisms have been elucidated and reviewed, we will only elaborate the last two stages in this review [81–82].

### Biosynthesis of crocetin (**1**)

In higher plants, the cyclization of lycopene (**5**) by lycopene ε-cyclase (ε-LCYC) and β-LCYC is a critical branch point in carotenoid biosynthesis. In one route, β-LCYC catalyzes the formation of the β-rings at both ends of **5**, leading to the formation of β-carotene (**6**). In the other branch, both ε-LCYC and β-LCYC are involved in introducing one β- and one ε-ring into **5** to form α-carotene. β-Carotene (**6**) is hydroxylated by β-carotene hydroxylase (BCH) to form **7**. Zeaxanthin (**7**) possesses strong antioxidative activity and is efficacious in preventing macular degeneration, cataracts, and cardiovascular diseases [83]. Many microorganisms, such as bacteria, cyanobacteria, and microalgae, can also produce **7** [84]. The biosynthesis of **7** in bacteria is analogous to that in plants. The difference is that in bacteria, **7** is formed through a single hydroxylation step of β-carotene (**6**), but it requires two hydroxylation steps in plants [84].

In the crocin biosynthetic pathways, lycopene (**5**), β-carotene (**6**), and zeaxanthin (**7**) are cleaved by different CCDs to form crocetin dialdehyde (**8**). *Cs*CCD2 from *C. sativus* could break the 7,8 and 7',8' double bonds of **7** to yield **8** and 3-OH-β-cyclocitral (**9**). ALDH then catalyzes the conversion of **8** into crocetin (**1**). Notably, *Gj*CCD4a from *G. jasminoides* catalyzes the cleavage of zeaxanthin (**7**), lycopene (**5**), and β-carotene (**6**) to generate **8** and conversion of **8** into **1** [85].

#### Biosynthesis of crocins

The late steps in crocin biosynthesis are the glycosylation of crocetin (**1**) by various UGTs [86]. The crocin biosynthetic pathways in *G. jasminoides* have been characterized in detail, and several *Gj*UGTs were identified. Attributed to different substrate specificities, the glucose or gentiobiose moieties are attached to the terminal carboxyl groups of **1** sequentially or simultaneously. Besides *Gj*UGTs, a few UGTs from *C. sativus* were also characterized. In addition to **1**, **9** is also glycosylated to form picrocrocin (**10**), which is further transformed into safranal (**12**) by β-glucosidase [87].

#### Key enzymes in crocin biosynthetic pathways

**CCDs:** Crocin biosynthesis initiates with the catalytic cleavage of the C‒C double bond by CCDs at various sites of the carotenoid skeleton. The cleavage generates a carbonyl group at the end of the formed carotenoid derivatives [88]. The CCD family can be categorized into the CCD subfamily and the 9-*cis*-epoxycarotenoid dioxygenase (NCED) subfamily. Five enzymes have been identified within the CCD subfamily: CCD1, CCD2, CCD4, CCD7, and CCD8. In animals, CCDs are involved in retinoid biosynthesis. In plants, CCD1 and CCD7 cleave the 9,10-double bond of carotenoids, while NCEDs cleave the 11,12-double bond [89]. Rubio et al. identified four CCDs, *Cs*CCD1a, *Cs*CCD1b, *Cs*CCD4a, and *Cs*CCD4b, from *C. crocus*. The expression patterns of *Cs*CCD4a and *Cs*CCD4b were associated with the accumulation of β-ionone during stigma development. However, no enzyme involved in zeaxanthin (**7**) cleavage was identified. Subsequently, *Cs*CCD2 was discovered in the cytoplasm alongside CCD1. *Cs*CCD2 was expressed at the early stage of stigma development, consistent with the accumulation of **1** initially. Sequence alignment of various CCDs from *C. crocus* and *Arabidopsis thaliana* revealed that *Cs*CCD2 belongs to a distinct branch within the CCD family. In vitro experiments confirmed that *Cs*CCD2 catalyzed the cleavage of **7** between the 7,8- and 7',8'-double bonds, resulting in the formation of crocetin dialdehyde (**8**) and 3-OH-β-cyclocitral (**9**) [90]. Recently, Liang et al. designed a variant of *Cs*CCD2 that exhibits broader substrate specificity and higher catalytic efficiency through computational modeling. Heterologous expression of the S323A mutant of *C*sCCD2 in yeast led to a 4-fold enhancement in the production of **1**, with a crocetin (**1**) titer of 107 mg/L in a 5-liter fed-batch fermentation. The *Cs*CCD2(S323A) mutant could also catalyze the conversion of β-carotene (**6**) to **1** [91]. *Bd*CCD4.1 and *Bd*CCD4.3, the homologs of CsCCD2 from *B. davidii*, were discovered by bioinformatic analysis. In vitro and in vivo experiments demonstrated that these two enzymes could cleave zeaxanthin (**7**), but the enzyme activity was lower than *Cs*CCD2 [2,92].

*Bo*CCD4-3 from *Bixa orellana* was revealed to cleave various carotenoids, lycopene (**5**), β-carotene (**6**), and zeaxanthin (**7**), to form crocetin dialdehyde (**8**) by in vitro assay [93–94]. In contrast, *Cs*CCD2, *Bd*CCD4.1, and *Bd*CCD4.3 could only cleave zeaxanthin (**7**) [95]. Xu et al. identified *Gj*CCD4a from *G. jasminoides*. Through prokaryotic expression and in vitro characterization, *Gj*CCD4a was found to cleave the 7,8-/7',8'-double bonds of lycopene (**5**), β-carotene (**6**), and zeaxanthin (**7**). Further investigation revealed that *Gj*CCD4a originated from tandem duplication events during genome evolution [96]. When expressed in the callus of *Nicotiana tabacum*, *Gj*CCD4a exhibited a higher efficiency in carotenoid cleavage than *Cs*CCD4a [85].

**ALDHs:** ALDH utilizes NAD^+^ or NADP^+^ as cofactor to catalyze the oxidation of acetaldehyde into carboxylic acids. In 2018, Demurtas et al. identified six ALDHs from *C. sativus* [97]. Among them, *Cs*ALDH3I1 oxidized crocetin dialdehyde (**8**) to crocetin (**1**). Subcellular localization and transgene expression analysis showed that *Cs*ALDH3I1 is located in the endoplasmic reticulum [97]. Gómez-Gómez et al. screened four genes (*CsALDH3898*, *CsALDH20158*, *CsALDH54788*, and *CsALDH11367*) from *C. sativus* and showed that all four enzymes could convert crocetin dialdehyde (**8**) into crocetin (**1**) [98]. Subsequently, Tan et al. used *Cs*ALDH3 to construct an engineered *Saccharomyces cerevisiae* strain for the heterologous production of crocetin (**1**) [99]. In 2020, Xu et al. conducted a multiomics analysis of 19 ALDH genes from *G. jasminoides*. Among them, *GjALDH2C3* was highly expressed in the fruit and flower of *G. jasminoides* and showed a coexpression pattern with *Gj*CCD4a [96]. Subsequently, Diretto et al. identified *Bd*ALDH from *B. davidii* and confirmed that it was able to convert crocetin dialdehyde (**8**) into crocetin (**1**) in vitro [100]. *Bd*ALDH and *Cs*ALDH3I1 belong to the ALDH3 family, one of the most diverse groups of the ALDH superfamily.

**UGTs:** Glycosylation of crocetin (**1**) improves the solubility significantly [101–102]. Moraga et al. cloned two UGT genes, *CsUGT2* and *CsUGT3*, from the stigmas of *C. sativus* and expressed them in *E. coli*. In vitro experiments showed that *Cs*UGT2 could glycosylate crocetin (**1**), crocin-V (**2e**), and crocinIV (**2d**) [86]. Demurtas et al. revealed that *Cs*UGT74AD1 could convert crocetin (**1**) into crocin-I (**2a**) and crocin-II (**2b**). Through subcellular localization analysis, they proposed that the biosynthesis of crocins initiates in the plastids. The metabolites are transported to the endoplasmic reticulum and cytoplasm and are stored in the vacuole. Moreover, they confirmed that the ABC transporter is involved in crocin accumulation in the vacuole within the stigmas of *C. sativus* [97]. López-Jimenez et al. screened the *UGT91P3* gene from *C. sativus* and showed that coexpression of *Cs*UGT74AD1 and *Cs*UGT91P3 in *N. benthamiana* produced various crocins. These results confirmed that *Cs*UGT91P3 plays a pivotal role in the biosynthesis of crocins in *C. sativus* [103].

Nagatoshi et al. identified *Gj*UGT75L6 and *Gj*UGT94E5 from *G. jasminoides*. *Gj*UGT75L6 could catalyze the glycosylation of crocetin (**1**) to produce crocetin glycosyl esters **2c** and **2e**, and *Gj*UGT94E5 catalyzed downstream glycosylation reactions [104]. Unexpectedly, the expression patterns of these two UGTs did not align with the accumulation of crocins, suggesting that additional enzymes may participate in crocin biosynthesis in *G. jasminoides*. Xu et al. screened 173 *Gj*UGTs from *G. jasminoides*. Through coexpression analysis with *GjCCD4a*, *Gj*UGT74F8 and *Gj*UGT94E13 were identified [105]. Specifically, *Gj*UGT74F8 catalyzed the conversion of crocetin (**1**) into crocin-V (**2e**) and crocin-III (**2c**), as well as the conversion of crocin-IV (**2d**) to crocin-II (**2b**). On the other hand, *Gj*UGT94E13 could convert crocin-II (**2b**) and crocin-III (**2c**) to crocin-I (**2a**) [105]. Diretto et al. screened *Bd*UGT74BC1 and *Bd*UGT74BC2 from *B. davidii* and found that both enzymes could glycosylate crocetin (**1**) to yield crocin-I (**2a**) and crocin-II (**2b**). They also isolated *Bd*UGT94AA3 from the flower of *B. davidii* and identified a role in the conversion of crocin-III (**2c**) into crocin-IV (**2d**) [100]. Ding et al. discovered two UGTs, Bs-GT and Bc-GTA, from *Bacillus subtilis*168 and *Bacillus cereus* WQ9-2, respectively. Bs-GT and Bc-GTA both exhibited UGT activity towards crocetin (**1**) [106].

### Heterologous production of crocins

Crocetin (**1**) and crocins have shown great value in the food, cosmetic, and pharmaceutical industries. However, the insufficient supply of crocins impedes the applications. Various strategies have been utilized to construct engineered microorganisms for the large-scale production of crocetin (**1**) and crocins (Table 2).

**Table 2:** Heterologous production of crocetin (**1**) and crocins.

host	production strategy	product	titer or yield	Reference

*S. cerevisiae*	combining and screening of *BCH-CCD*, overexpression of ALD*,* Δ*CIT2*, and *Ps*BCH*-Cs*CCD2	**1**	12.43 ± 0.62 mg/L in fermenter	[107]
*S. cerevisiae*	introduction of CrtE, CrtYB, CrtI, and CrtZ in yeast, overexpression of *Cs*CCD2-*Cs*ALDH, overexpression of *Ps*BCH-*Cs*CCD2	**1**	139.67 ± 2.24 μg/g DCW	[108]
*E. coli*	introduction of *Cs*CCD2*-Gj*UGT94E15*-Gj*UGT75L6*-Trc*ALD8	**1**	4.42 mg/L	[109]
*E. coli*	adjusting the molar ratio of *Cs*CCD2 and *Syn*ALD	**1**	1.48 mg/L/h	[110]
*E. coli*	introduction of *Cs*CCD2*-Gj*UGT94E15*-Gj*UGT75L6*-Trc*ALD8, YjiC*,* YdhE*,* and YojK from *B. subtilis*	**2e**	**—**	[109]
*E. coli*	introduction of *Gj*UGT94E13*-Gj*UGT74F8, overexpression of PGM and GalU	crocins	**—**	[105]
*N. benthamiana*	introduction of *Cs*CCD2L and *Pa*CrtB	crocins	3.493 mg/g DCW	[92]
*N. benthamiana*	introduction of *Cs*CCD2L*-Os*BCH*-Tp*CartB	crocins	1.61 mg/g	[85]
*N. tabacum* and *N. glauca*	introduction of *At*OrMut*-Br*CrtZ*-Cs*CCD2L	crocins	400 μg/g DW	[111]
*S. lycopersicum*	introduction of *Cs*CCD2L*-Gs*UGT709G1*-Cs*UGT2	crocins	14.48 mg/g	[112]
*N. benthamiana*	overexpression of *Cs*ABCC4a*-Cs*CCD2	crocins	**—**	[113]
*N. benthamiana*	overexpression of *Cs*CCD2 in the zeaxanthin (**7**)-producing strain	crocins	800 µg/g DW	[97]
*N. benthamiana*	expression of *GjCCD4a-GjALDH2C3-GjUGT74F8-GjUGT94E13* multigene expression vector	crocins	78 µg/g FW	[114]

#### Heterologous synthesis of crocetin (**1**)

Chai et al. first achieved the heterologous production of crocetin (**1**) in *S. cerevisiae* [107]. Through combining and screening the BCHs, CCDs, and ALDHs and optimizing the fermentation conditions, 1219 μg/L and 6278 μg/L of crocetin (**1**) was produced in the shake flask and a 5 L fermenter, respectively. Followed by enhancing acetyl-CoA supply, overexpressing *Ps*BCH-*Cs*CCD2, and optimizing the fermentation medium, the yield of crocetin (**1**) reached 12.43 ± 0.62 mg/L in a 5 L bioreactor [115]. While CCD has a higher catalytic activity at low temperature, the biosynthesis of zeaxanthin (**7**) is more suited to a higher temperature. As such, Liu et al. used CRISPR-Cas9 to create a temperature-responsive strain of *S. cerevisiae* for crocetin (**1**) production. By increasing the copy number of *CCD2* and *ALDH*, the conversion of zeaxanthin (**7**) was increased by 77% [108]. Wang et al. screened the *ALD* genes from *C. sativus* and *Neurospora crassa* and showed that the strain expressing *Nc*ALD8 could produce 4.42 mg/L of crocetin (**1**) [109]. In addition to the de novo biosynthesis of crocetin (**1**), Shan et al. reported the biotransformation of crocetin (**1**) from zeaxanthin (**7**). When the 3-OH-β-apo-8'-carotenoic acid (3-HACA) byproduct was eliminated by adjusting the molar ratio of *Cs*CCD2-M1 and *Sy*nALD in the system, the yield of crocetin (**1**) reached 5.92 mg/L with a conversion rate of 80.8% [110].

#### Heterologous biosynthesis of crocins

The heterologous production of crocins has been achieved in various hosts, including *E. coli*, *N. benthamiana*, and *S. lycopersicum.* Wang et al. introduced YjiC and YojK, two UGTs from *B. subtilis* into a crocetin (**1**)-producing *E. coli* strain to realize the biosynthesis of crocin-V (**2e**) [109]. Pu et al. biosynthesized different crocins by introducing *Gj*UGT74F8 and *Gj*UGT94E13 from *G. jasminoides* [105]. However, the titers of the produced crocins were not reported. Heterologous biosynthesis of crocins was also attempted in *Nicotiana tabacum* and *Lycopersicon esculentum* [85]. Because *Cs*CCD2L exhibited high CCD activity against zeaxanthin (**7**), Martí et al. used a virus-driven transduction system to transfer *CsCCD2L*, *PaCrtB* (the gene encoding the carotenoid synthase from *Pantea ananatis*), and *BCH2* from *C. sativus* into *N. benthamiana*. The content of crocins in the transgenic *N. benthamiana* reached 3.493 ± 0.325 mg/g dry weight [92]. Zheng et al. introduced *GjCCD4a*, *PSY*, and *BCH* into *N. benthamiana* and achieved a crocin content of 1.61 mg/g in the leaves [85]. It was reported that CrtZ catalyzes the rate-limiting reaction in zeaxanthin (**7**) biosynthesis, and the ORANGE protein regulates PSY expression to increase carotenoid accumulation. Ahrazem et al. coexpressed *AtOrMut* from *A. thaliana*, *BrCrtZ* from a *Brevundimonas* sp., and *CsCCD2L* into *N. tabacum* and achieved a crocin content of 400 μg/g (dry weight) in the engineered plant. Compared to transgenic *N. tabacum* that expressed *Cs*CCD2L alone, the crocin yield was increased 3.5-fold [111]. Ahrazem et al. also introduced *CsCCD2L*, *CsUGT709G1*, and *CsUGT2* into *L. esculentum* to produce crocins. The transgenic plants exhibited a dry weight yield of 14.48 mg/g for crocins and 2.92 mg/g for picrocrocin (**10**), representing the highest yield of crocins in transgenic plants [111–112]. Augustine et al. characterized the transporter ABCC4a for crocin transportation through transcriptomic and metabolomic analysis. Coexpression of ABCC4a with CCD2 in the leaves of *N. benthamiana* increased the yield of crocins [113].

Crocin-I (**2a**) and crocin-II (**2b**) are the main active crocin components, but the proportion of these two compounds in the total crocin content was usually low in heterologous hosts. Xie et al. introduced a multigene expression vector (*GjCCD4a*-*GjALDH2C3*-*GjUGT74F8*-*GjUGT94E13*) into *N. benthamiana* by using the fusion and 2A polypeptides strategy and obtained a crocin yield of 78,362 ng/g (fresh weight). The content of crocin-I (**2a**) and crocin-II (**2b**) accounted for 99% of the total crocin content [114]. The supply of UDP-glucose is a rate-limiting step in crocin biosynthesis. Glucose is first phosphorylated to generate glucose-6-phosphate by hexokinase and then converted into glucose-1-phosphate by phosphoglucomutase (PGM1). Afterwards, UTP-glucose-1-phosphate uridylyltransferase (GalU) converts glucose-1-phosphate into UDP-glucose. Pu et al. introduced *Gj*UGT74F8 from *G. jasminoides* into an *E. coli* strain overexpressing *PGM* and *GalU* and achieved the production of crocins [105].

## Conclusion

Crocins possess the characteristic polyene core structure of carotenoids and exhibit broad pharmacological properties. They are promising drugs for treating cardiovascular and liver diseases. Despite numerous pharmacological properties, the exact mechanisms of action of crocins remain elusive. The therapeutic potential of crocins is impeded by the limited bioavailability and rapid clearance in vivo. Only a few plants can produce crocins, and the content of crocins in these plants is very low. Due to the numerous chiral centers, the total synthesis of crocins is challenging. Therefore, heterologous biosynthesis of crocins utilizing the synthetic biology strategy holds great potential to solve the supply issue of crocins.

Significant progress has been made in the heterologous production of carotenoids in microorganisms in recent decades. The biosynthetic pathways of crocins have been extensively elucidated, and many enzymes in these pathways have been identified. The CCDs, ALDHs, and UGTs are the key players in crocin biosynthesis. Empowered by the genomic, transcriptomic, and metabolomic analysis and biochemical characterization, many such enzymes have been identified. Some proteins, exemplified by *Gj*CCD4a and *Gj*UGT94E13, exhibit broad substrate promiscuity and high catalytic activity. Screening of novel enzymes for crocin biosynthesis and the directed evolution of known enzymes should facilitate the high-titer production of the key intermediates **5–7** etc. and the efficient conversion of these precursors into crocetin (**1**) and crocins.

In conclusion, crocins are high-value plant natural products, and their biosynthetic pathways have been extensively studied. The construction of efficient hosts for the heterologous production of crocins will be a practical solution for the industrial production of these important chemicals.

## Data Availability

Data sharing is not applicable as no new data was generated or analyzed in this study.
